# T-Cell Development in Early Partially Decapitated Chicken Embryos

**DOI:** 10.1155/1995/81462

**Published:** 1995

**Authors:** Javier Moreno, Angeles Vicente, Alberto Varas, Agustín G. Zapata

**Affiliations:** 1 Centro Nacional de Microbiología Virología Inmunología Sanitarias Instituto de Satud Carlos III Majadahonda Madrid 28220 Spain; 2 Department of Cell Biology Faculty of Biology Complutense University Madrid 28040 Spain

**Keywords:** T-cell development, thymus, chicken embryos

## Abstract

We have evaluated the immunohistological and cytofluorometric changes that occur in the thymus of chicken embryos partially decapitated at 33-38 hr of incubation (DCx embryos) in an attempt to analyze possible neuroendocrinological influences on T-cell differentiation and, indirectly, the ontogeny of the so-called neuroendocrine-immune network. The thymus of DCx embryos shows important variations that profoundly and selectively affect different T-cell subsets, but not the nonlymphoid cell components of thymic stroma. These modifications include the accumulation of cell precursors, mainly DN (CD4^-^ CD8^-^) cells and immature CD8^high^ CD4^-^ cells, which expand but do not differentiate, resulting in an extreme decline of both DP (CD4^+^ CD8^+^) cells and TcR c-expressing cells. Accordingly, both subcapsulary and outer cortex increase in size, whereas the deep cortex and principally the thymic medulla almost disappear in DCx embryos. In contrast, other T-cell subsets of DCx embryos, largely CDg^glow^CD4^-^ cells and TcR *γδ*-expressing cells do not undergo significant variations throughout thymic ontogeny.


*Developmental Immunology*, 1995, Vol 4, pp. 211–226
Reprints available directly from the publisher
Photocopying permitted by license only

© 1995 Harwood Academic Publishers GmbH
Printed in Singapore

### T-Cell Development in Early Partially Decapitated Chicken Embryos

JAVIER MORENO,[†] ANGELES VICENTE,[‡] ALBERTO VARAS,[‡] and AGUSTÍN G. ZAPATA*[‡]

[†]*Centro Nacional de Microbiología, Virología e Inmunología Sanitarias, Instituto de Salud Carlos III, 28220 Majadahonda, Madrid, Spain*

[‡]*Department of Cell Biology, Faculty of Biology, Complutense University, 28040 Madrid, Spain*

We have evaluated the immunohistological and cytofluorometric changes that occur in the thymus of chicken embryos partially decapitated at 33–38 hr of incubation (DCx embryos) in an attempt to analyze possible neuroendocrinological influences on T-cell differentiation and, indirectly, the ontogeny of the so-called neuroendocrine-immune network. The thymus of DCx embryos shows important variations that profoundly and selectively affect different T-cell subsets, but not the nonlymphoid cell components of thymic stroma. These modifications include the accumulation of cell precursors, mainly DN ($CD4^-CD8^-$) cells and immature $CD8^{low}CD4^-$ cells, which expand but do not differentiate, resulting in an extreme decline of both DP ($CD4^+CD8^+$) cells and TcR αβ-expressing cells. Accordingly, both subcapsulary and outer cortex increase in size, whereas the deep cortex and principally the thymic medulla almost disappear in DCx embryos. In contrast, other T-cell subsets of DCx embryos, largely $CD8^{high}CD4^-$ cells and TcR γδ-expressing cells do not undergo significant variations throughout thymic ontogeny.

KEYWORDS: T-cell development, thymus, chicken embryos.

### INTRODUCTION

Increasing evidence in recent years has verified the intimate relationship between mammalian neuroendocrine and immune systems (Besedovsky et al., 1985a, 1985b; Hadden, 1992). On the one hand, various endocrine hormones and factors have immunoregulatory capacities and, on the other hand, cytokines produced by the immune system modulate numerous endocrine functions (Weigent and Blalock, 1987; Covelli et al., 1992). In this circuit, the thymus gland occupies a pivotal position and these relationships between the thymus and the neuroendocrine system could be established early during development. Pioneer studies described thymic regression in hypophysectomized rats (Smith, 1930) and Snell-Bagg and Ames dwarf mice with important deficiencies in prolactin (PRL), growth hormone (GH), and other neuroendocrine mediator production, and show early thymic involution with diminished thymocyte production and cell depletion in both bone marrow and peripheral lymphoid organs (Baroni, 1967; Baroni et al., 1967; Duquesnoy, 1972). More recent studies have confirmed these results and also indicate that DP ($CD4^+CD8^+$) thymocytes are cell targets for a lack of both GH and PRL (Cross et al., 1992; Murphy et al., 1992a, 1992b).

Despite these data, obvious difficulties for *in vivo* manipulation of mammalian fetuses have left many aspects of the ontogeny of the neuroendocrine-immune network unresolved. The chicken embryo, however, allows easy and precise access during early ontogeny, providing a suitable model system in which to focus this problem. Furthermore, birds also have a neuroendocrine-immune network (Glick, 1984) and the development of the chicken immune system is very similar to that of mammals (Vainio and Lassila, 1989; Chen et al., 1991; Cooper et al., 1991).

Jankovic and colleagues used an experimental model that stemmed from classical endocrinology, which involved the removal of major endocrinology centers (i.e., hypothalamus, pituitary gland, and pineal system) by partial decapitation of 33–38 hr embryonic chickens (DCx embryos), to demonstrate a delayed development of the lymphoid system in

* Corresponding author.

DCx embryos, that they attributed to an imbalance in neuroendocrine activity (Jankovic et al., 1978, 1981, 1982). More recently, we confirmed and extended these results analyzing the morphometrical and ultrastructural changes occuring in the thymus of DCx embryos (Herradón et al., 1991). Our study demonstrated a reduced size of the thymus gland with imbalanced development of cortex and medulla, accumulation of precursor cells in enlarged connective tissue trabeculae, intrathymic granulopoiesis, and hypertrophied epithelial cells. However, we did not resolve the issue of identifying T-cell subsets specifically affected by this experimental procedure. Remarkably, Jankovic et al. (1978) described no changes in the percentage of T cells identified by a specific antiserum, and recently Johnson et al. (1993), despite reporting important changes in the thymic weight of hypophysectomized adult chickens, did not find substantial modifications in the proportion of either $CT1^+$, $CD4^+$, or $CD8^+$ thymocytes. They reported, however, a decreased expression of both CD4 and CD8 surface markers on DP ($CD4^+CD8^+$) thymocytes and imbalance of the $CD4^+/CD8^+$ cell ratio in peripheral blood.

Hence, in the present study, we combine immunohistochemistry and flow cytometry to phenotypically identify the lymphoid and nonlymphoid thymic-cell subpopulations affected by early partial decapitation and to quantify the nature of their changes throughout ontogeny of DCx chicken embryos as an indirect way to analyze the ontogenetical appearance of the neuroendocrine-immune network.

### RESULTS

### Characterization of DCx Embryos

The viability was lower in DCx embryos than in Sham-DCx ones. Most deaths occurred at the first week of incubation, and from day 11 onward, embryos did not survive after day 17 of incubation. As previously described by Herradón (1987), Sham-DCx embryos were able to hatch normally. DCx embryo weight was also lower than in Sham-DCx embryos, but differences were only significant from day 11 and increased throughout development. As a consequence of the surgical procedure, DCx embryos lacked eyes and upper beak, presenting edema in the neck and dark skin.

### Haemopoiesis in the Aortic Mesenchyme

Because lymphohaemopoietic cell precursors have been described in the paraortic mesenchyme from 3–7-day-old embryonic chicken (Dieterlen-Lievre, 1992), we analyzed by light microscopy presumable changes in this area of DCx embryos on days 4 and 7 of incubation. Aortic and paraortic regions presented a similar aspect in both DCx and Sham-DCx embryos, although a quantitative, morphometrical study of their cell content was not carried out. At 4 days, groups of basophilic cells were detected near the lumen of the vessel on both sides of the ventral region of the dorsal aortic wall (Figs. 1 and 2). Moreover, similar cells, some in mitosis, appeared isolated throughout the mesenchyme surrounding the aorta. They resembled, in aspect and location,

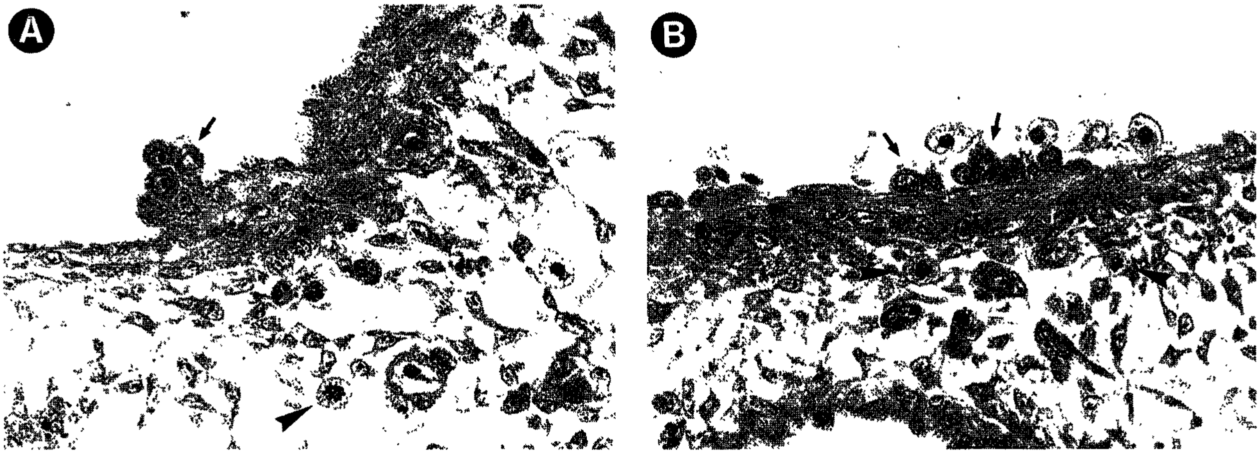

FIGURE 1. Wall of the dorsal aorta of 4-day-old (A) DCx and (B) embryos Sham-DCx. Groups of cells (arrows), some of them in division (arrowheads), present near the vessel lumen or in the mesenchyme surrounding it. ×250.

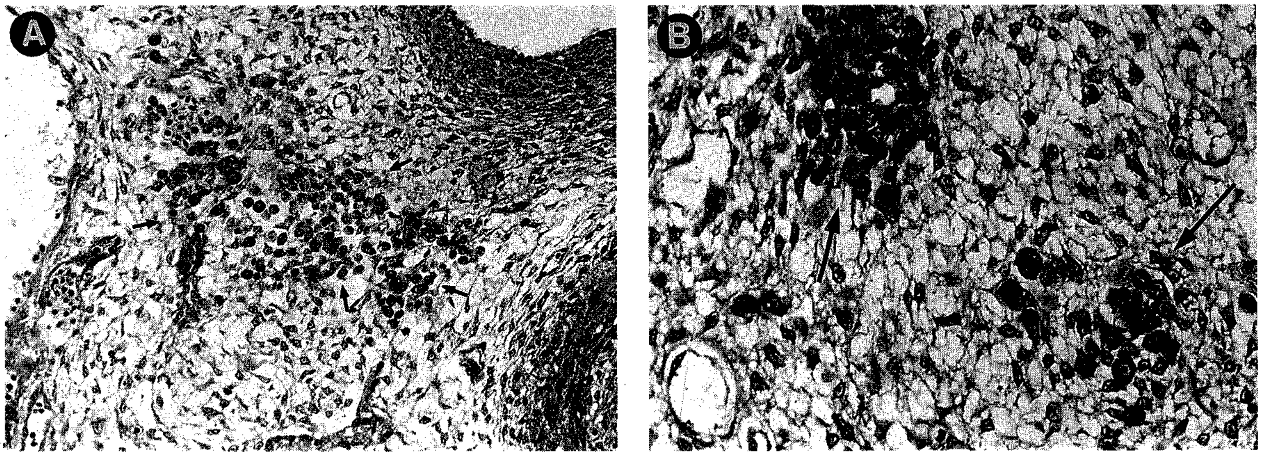

FIGURE 2. Paraortic foci (arrows) in the mesenchyme of a 7-day-old DCx (A, × 125) and Sham-DCx embryo (B, × 250).

those reported in these areas as early haemopoietic cell precursors (Dieterlen-Lievre and Martin, 1981). On day 7, groups of cells that appeared in the paraortic mesenchyme (Fig. 2) were similar to the so-called paraortic foci described as being able to produce *in vitro* thymocyte (Cormier et al., 1986) or monocyte colonies (Cormier and Dieterlen-Lievre, 1988).

### Thymus Development

The thymus of DCx embryos showed a decreased size and delayed development that was more patent in the older embryos (Fig. 3A), compared to Sham-DCx embryos (Fig. 3B). These changes were reflected in the low number of cells yielded by the thymic-cell suspensions from DCx embryos and the high number of large blast cells it contained. Moreover, DCx thymus exhibited enlarged connective tissue trabeculae and it was difficult to clearly distinguish corticomedullary junction (Fig. 3A).

### Thymocytes

As shown in Fig. 4, the proportion of positive cells for pan T-cell markers, such as CVI-His-$C_7$ and CD28, increased from day 11 to 15 in both DCx and Sham-DCx embryos, but they were always lower in the former. Interestingly, whereas the percentage of these cells in Sham-DCx embryos increased continuously throughout ontogeny, in DCx embryos, it underwent a sharp decrease from day 15 of incubation (Fig. 4). In correlation, immunostaining of thymic sections from 17-day-old DCx embryos with

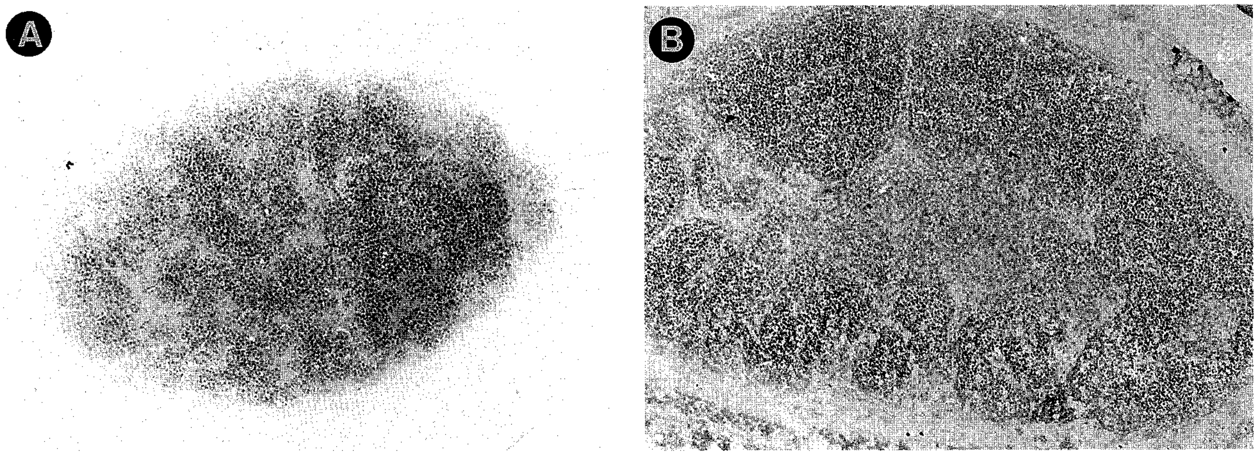

FIGURE 3. Section of a thymic lobe from a 15-day-old DCx (A) and Sham-DCx embryo (B). Note the smaller size of the former as well as the difficult to distinguish the cortex-medulla junction. × 62.

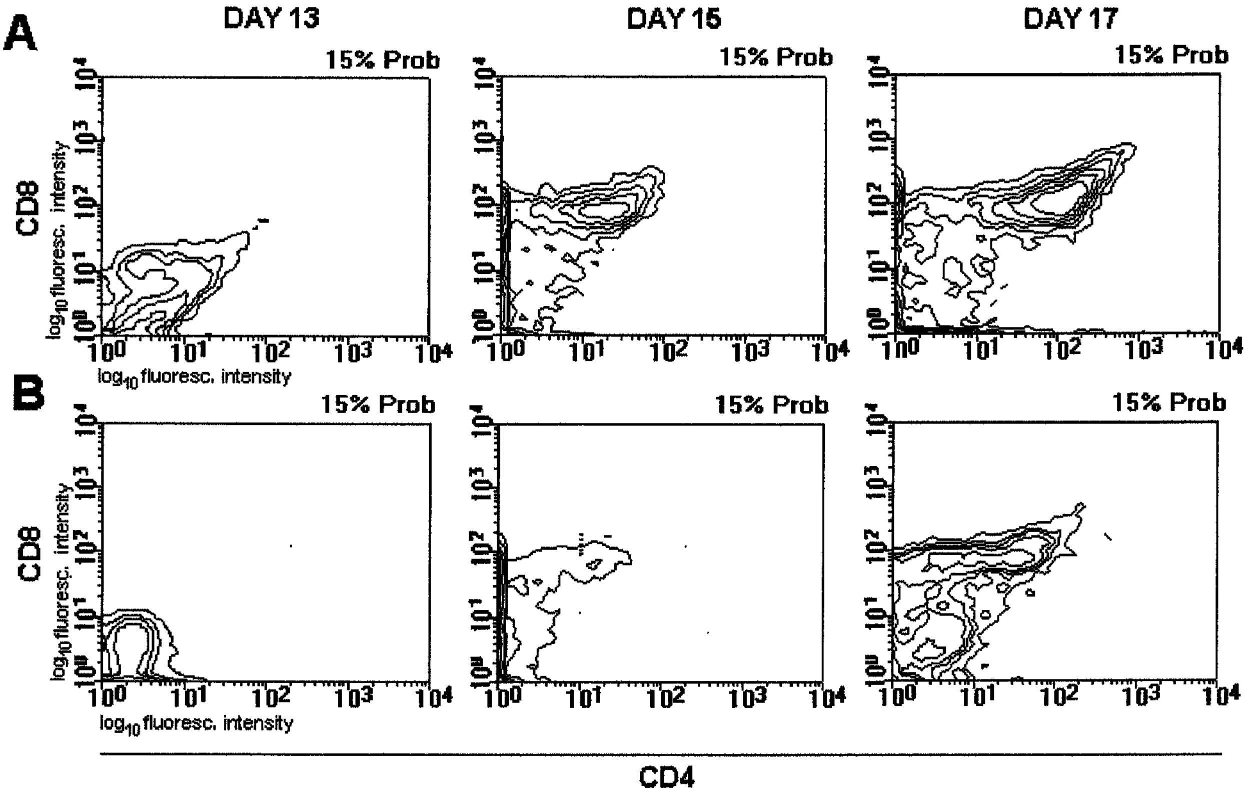

FIGURE 7. Expression of CD4 and/or CD8 throughout thymic ontogeny of Sham-DCx (A) and DCx embryos (B).

values at day 15 and 17 of incubation (Figs. 8 and 9), whereas the number of DN ($CD4^-CD8^-$) cells decreased in parallel to that observed in Sham-DCx embryos, although remaining significantly higher than the latter (Fig. 9). In summary, DCx embryos seemed to accumulate immature thymocytes, including both DN ($CD4^-CD8^-$) cells and $CD8^{low}$ cells (Fig. 9), resulting in a decrease in the proportion of DP ($CD4^+CD8^+$) cells present (Fig. 10).

The first TcR-expressing cells in both DCx and Sham-DCx embryos corresponded to γδ T cells, which were detected on day 13, increasing on day 15 to show some decline at 17 days (Fig. 11). No important differences were observed in the percentage of this T-cell population between DCx and control embryos (Fig. 11), appearing in both mainly in the thymic medulla (Fig. 12). In contrast, the first TcR αβ-expressing cells occurred in 15-day-old

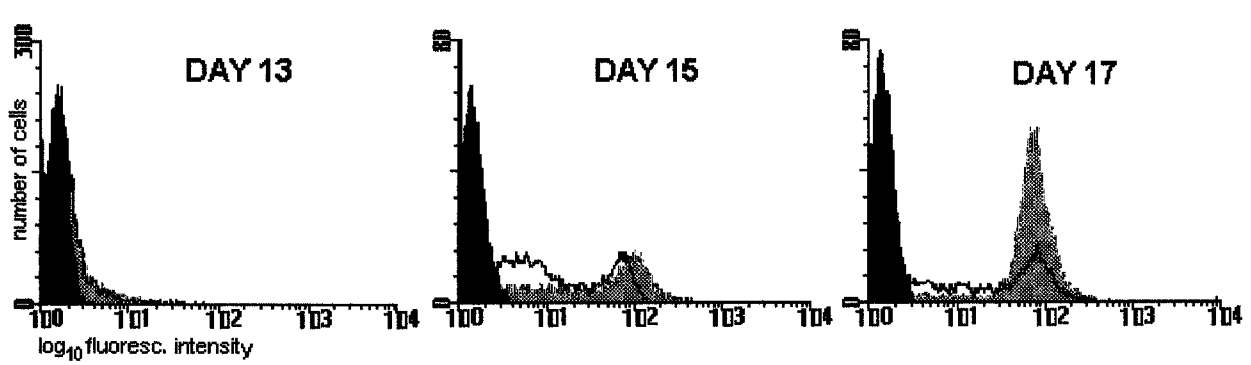

FIGURE 8. CD8 expression in thymocytes from Sham-DCx (shaded profile) and DCx embryos (black line) at different days of incubation. Negative control, black profile.

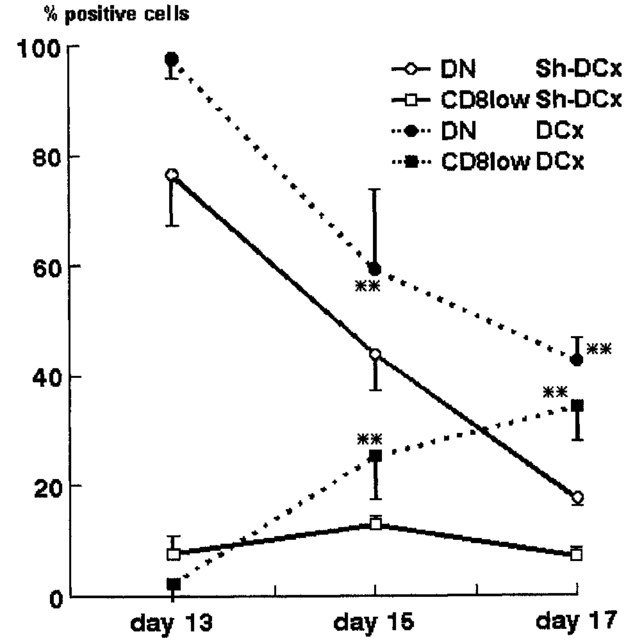

FIGURE 9. Percentages of DN (CD$^-$CD8$^-$) cells and immature SP CD8$^{low}$CD4$^-$ cells in DCx and Sham-DCx embryos; $p \leq 0.1$ and $p \leq 0.05$ significant differences are marked as * and **, respectively.

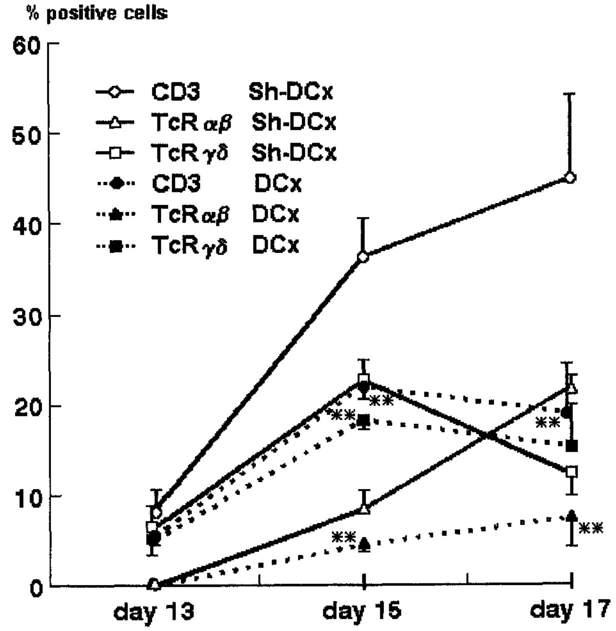

FIGURE 11. Percentages of CD3-positive cells, TcR αβ-expressing cells and TcR γδ-expressing cells in DCx and Sham-DCx embryos; $p \leq 0.1$ and $p \leq 0.05$ significant differences are marked as * and **, respectively.

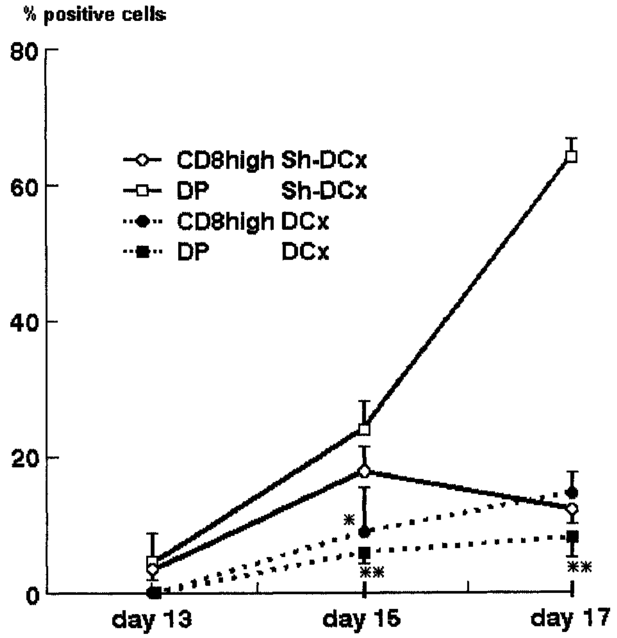

FIGURE 10. Percentages of DP (CD4$^+$CD8$^+$) cells and SP CD8$^{hi}$CD4$^-$ cells in DCx and Sham-DCx embryos; $p \leq 0.1$ and $p \leq 0.05$ significant differences are marked as * and **, respectively.

Sham-DCx embryos, increasing rapidly in the following developmental stage. At 15 days, when the flow cytometry detected only a few αβ T cells, the immunohistochemical study demonstrated numerous positive cells in the thymic cortex, suggesting that the molecule was predominantly expressed in the cell cytoplasm. In DCx embryos, the percentage of TcR αβ-expressing cells was significantly lower than that found in control Sham-DCx embryos, being difficult to identify immunohistologically on thymic sections (Fig. 11). The number of CD3-positive cells represented the addition of those that expressed the αβ and γδ T-cell receptor (Fig. 11), because CD3 is coexpressed in both T-cell lineages. Therefore, in DCx-embryos, their percentage was significantly lower than in control ones, with the majority of CD3$^+$ cells corresponding to those expressing the γδ T-cell receptor.

Analysis of the cell cycle demonstrated a higher number of cells in division in Sham-DCx embryos than in DCx ones at both 15 and 17 days of incubation (Fig 13). Remarkably, on day 15, while the dividing cells of control thymus were CD28$^+$ cells and the vast majority expressed also CD8, in

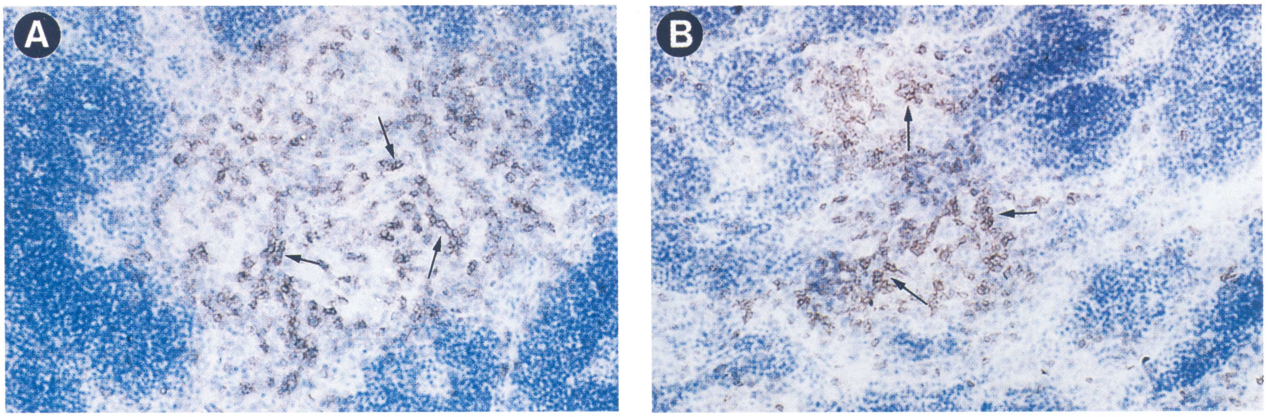

FIGURE 12. TcR $\gamma\delta$-expressing cells (arrows) in the thymic medulla from a 15-day Sham-DCx embryo (A) and a 17-day-old DCx embryos (B). $\times$ 125. See Colour Plate XIX.

DCx embryos, the majority of cells cycling were $CD8^-$ and to a lesser extent $CD28^-$ cells, indicating their condition as primitive cell precursors (Fig. 14). This situation was similar, although with a smaller difference, in 17-day-old embryos (Fig. 15).

**Stromal-Cell Components**

Apart from the previously mentioned decreased in thymus size in DCx embryos, no important differences were found between the thymic stromal-cell

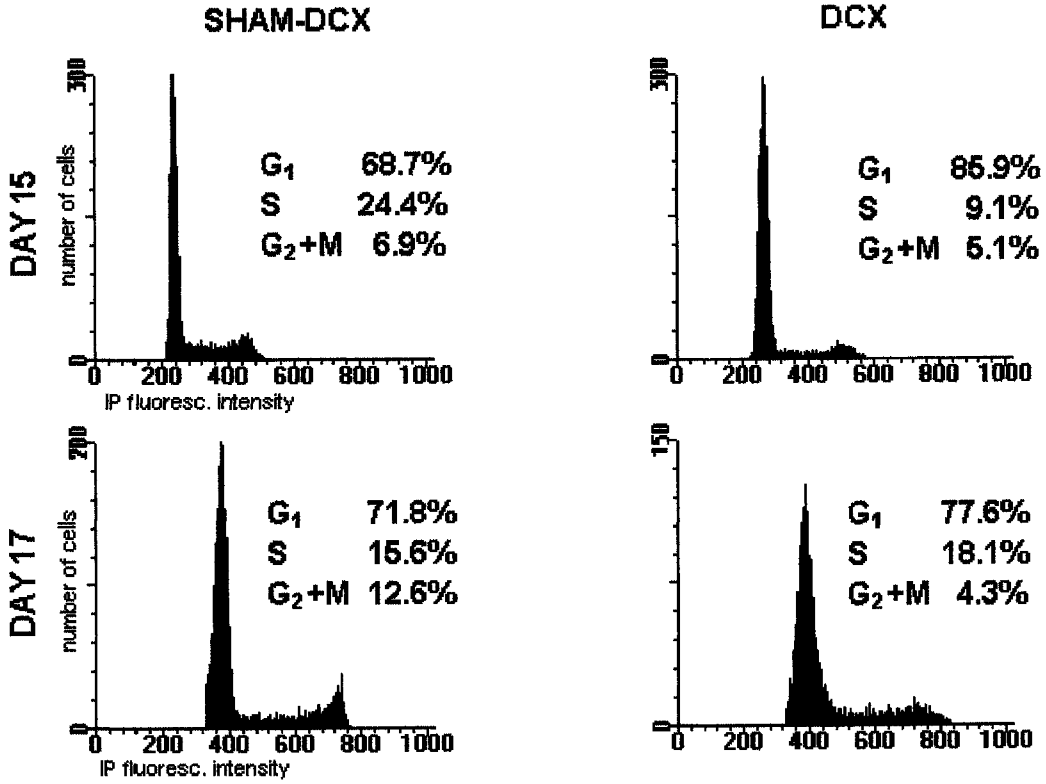

FIGURE 13. Cycling thymocytes from both 15- and 17-day-old Sham-DCx and DCx embryos.

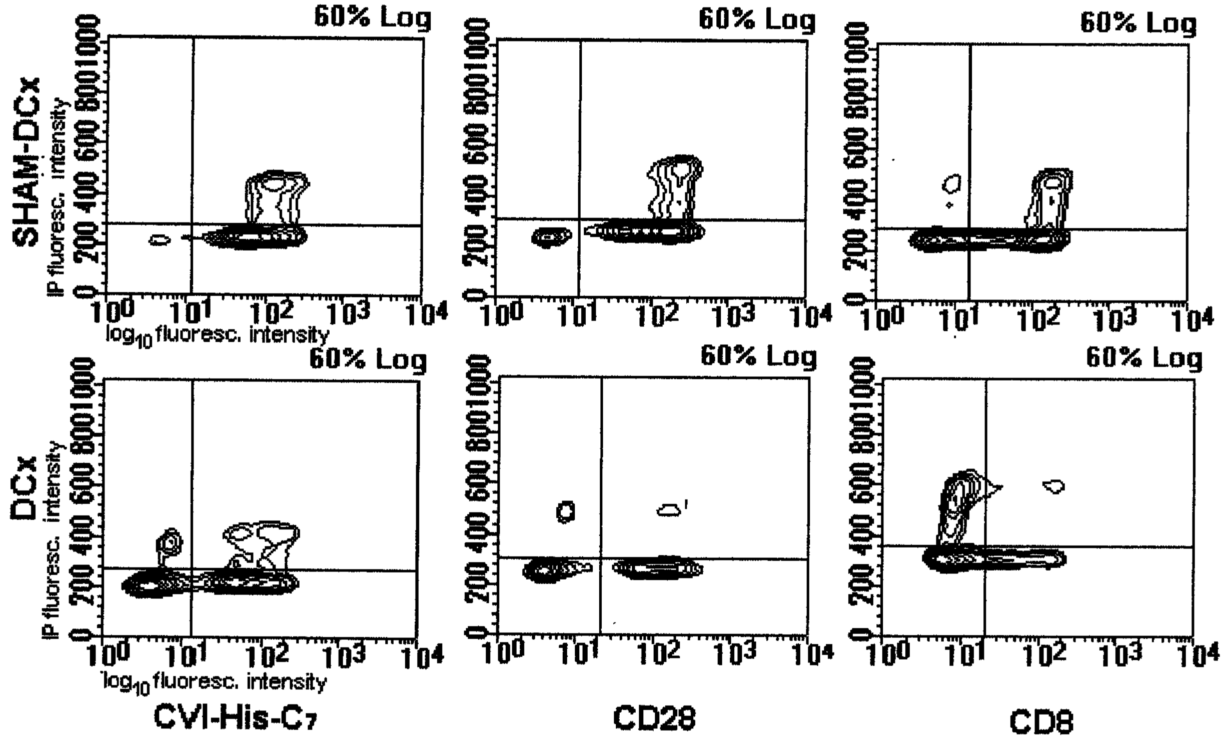

FIGURE 14. Expression of CVI-His-$C_7$, CD28, or CD8 on cycling thymocytes from 15-day-old Sham-DCx and DCx embryos.

components, including epithelial cells, macrophages, and interdigitating cells, of DCx and control Sham-DCx embryos.

MHC class II molecule-expressing cells (MYC-$16^+$) had already appeared in the thymus on day 11 of incubation, as well as MUI-$36^+$ macrophages and CVI-ChNL-$68.1^+$ cells, presumably corresponding to monocytes, macrophages, and interdigitating cells. In the following stages, the number of MYC-$16^+$ cells increased and the medullary region appeared totally stained with foci of positive cells in both the cortex and subcapsullary area (Fig. 16). The number of both MUI-$36^+$ and CVI-ChNL-$68.1^+$ cells increased more slowly, although always without significant differences between DCx and Sham-DCx embryos. In both cases, the positive cells predominated in the thymic medulla, with a few CVI-ChNL-$68.1^+$ cells scattered throughout the cortex (Fig. 17).

The immunological study using mAbs MUI-52, MUI-53, and MUI-55 specifically raised to cortical, medullary, and subcapsular/subtrabecular epithelium, respectively, revealed positive cells from day 15 of incubation. Stained-cell processes formed a network in the medulla (MUI-$53^+$ cells) and subcapsular area (MUI-$55^+$ cells), and positive cells occurred in the thymic cortex (MUI-$52^+$ cells). On day 17, the stained-cell processes formed a more extensive network. As shown in Fig. 18, no differences were found between the control and experimental thymuses, with a similar distribution pattern and extent of staining in both groups. Therefore, at least with the methodological approach, there were no apparently significant differences in the number, staining pattern, or location of the thymic stromal-cell components between DCx and Sham-DCx embryos.

### Endocrinological Background

The histological analysis of the thyroid (Fig. 19A), adrenal gland (Fig. 19B), and gonads (Figs. 19C and 19D) showed a normal development in Sham-DCx embryos, whereas in the DCx ones, they presented a clear delay in the growth that increased throughout incubation, confirming the lack of circulating hormones in these embryos previously demonstrated by Herradón (1987). The thyroid showed few, if any, follicles and none of them contained follicular fluid (Fig. 20A). The interrenal tissue was

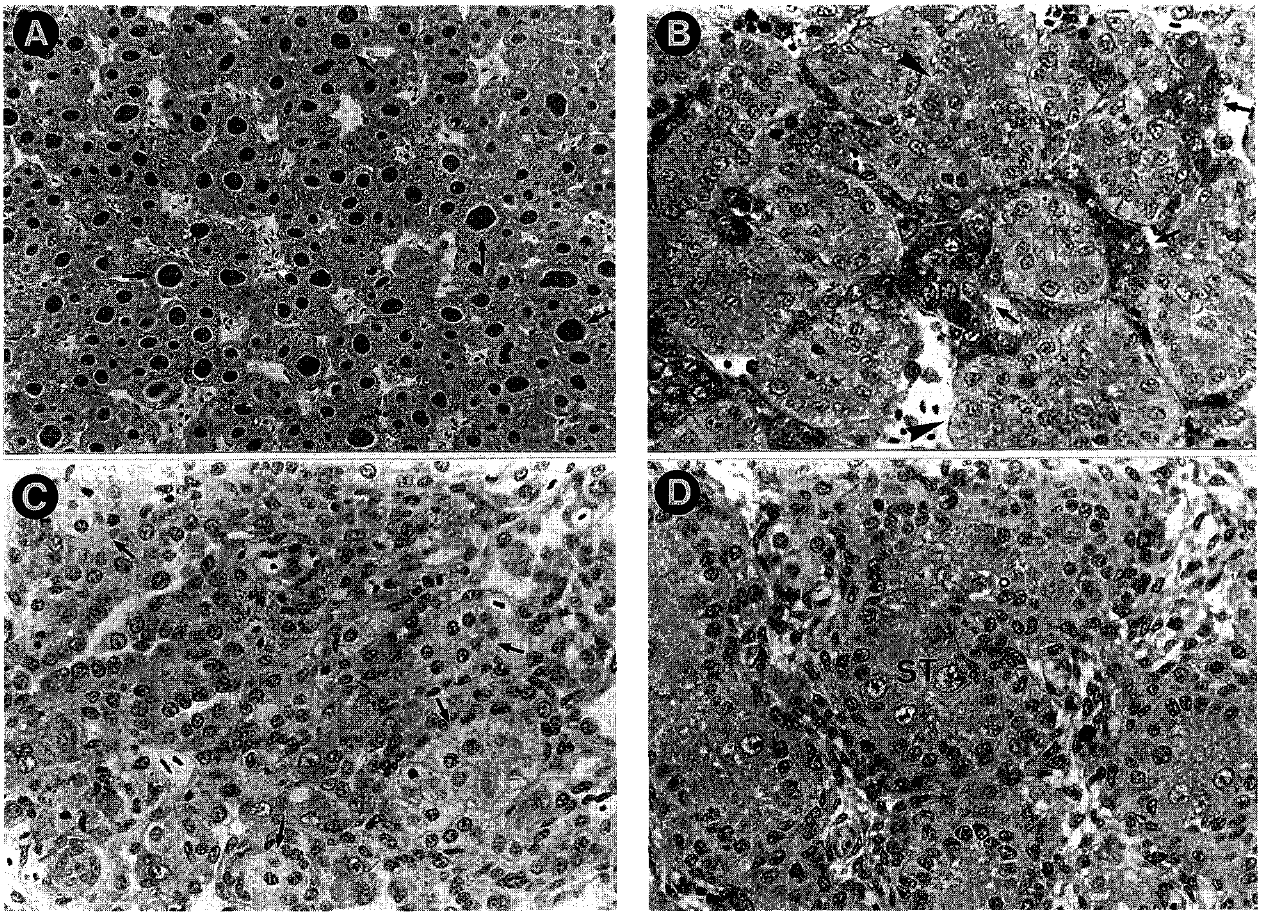

FIGURE 19. Normal histological features of the thyroid (A, × 125), adrenal gland (B, × 250), ovary (C, × 250), and testis (D, × 250) of 15-day-old Sham-DCx embryos. Note the presence of colloidal fluid in the thyroidal follicles (arrows) (A), chromaffin tissue (arrows), and cords of interrenal tissue (arrowheads) in the adrenal gland (B), groups of interstitial cells (arrows) in the ovary (C), and seminiferous tubules (st) in the testis (D).

In contrast, DCx-embryos, as a consequence of the early elimination of major neuroendocrine centers, show a low viability, little body growth, especially during the second half of incubation, and an important delay until the histological development of endocrine organs ceases, as previously reported by numerous authors on the same experimental model (Fugo, 1940; Vogel, 1957; Betz, 1967; Thommes et al., 1977; Woods et al. 1989). Likewise, histological changes observed during the thymic ontogeny of DCx-embryos, including decreased medullary development, enlarged connective tissue trabeculae, and so on, coincide totally with those reported by other authors (Jankovic et al., 1978, 1980, 1981, 1982; Micic et al., 1983; Herradón, 1991), whereas Fugo (1940) did not find reportable modifications in the thymus histology of DCx embryos.

There is no published evidence, however, on the effects of this experimental procedure on the ontogenetical development of intrathymic T-cell subsets. Jankovic et al. (1978), using a rabbit anti-chicken T-cell antiserum, did not observe important differences in the number of thymocytes, although histologically they reported, in agreement with our results, thymocyte depletion in DCx-embryos. In addition, Johnson et al. (1993) did not find changes in the percentage of either $CT1^+$, $CD4^+$, and $CD8^+$ thymocytes of hypophysectomized adult chickens, although, in our opinion, the model shows evident differences.

In contrast, both our immunohistological and flow cytofluorometric results support an important delay in the maturational process of the T-cell system of DCx-embryos. This results in an accumulation of

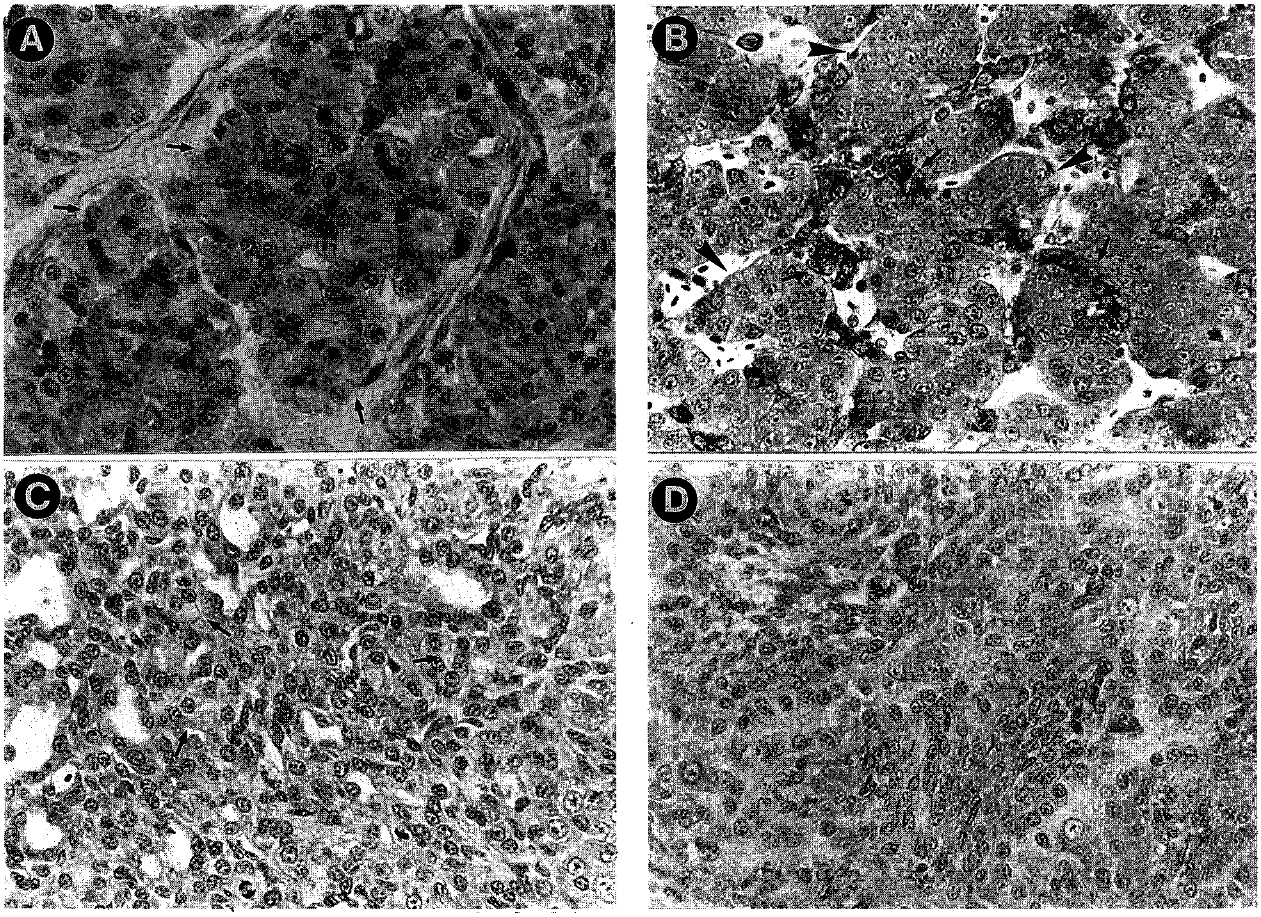

FIGURE 20. Lack of colloidal follicles in the thyroid of 15-day-old DCx embryos (A). A few chromaffin cells (arrows) and interrenal cells without signs of corticoid secretion (arrowheads) occur in the adrenal gland of 17-day-old DCx embryos (B). Small groups of interstitial cells (arrows) appear in the ovarian parenchyma of 17-day-old DCx embryos (C). Absence of seminiferous tubules in the testis of 17-day-old DCx embryos (D). ×250.

precursor cells not only in the enlarged thymic trabeculae, as previously described (Herradón, 1991), but also in the intrathymic subcapsulary cortex, which appears devoid of immunohistological staining of mAbs CVI-His-$C_7$, 2-4, CT8, 5-5, CT1, and MUI-83. On the other hand, this accumulation correlated well with increased number of both DN ($CD4^-CD8^-$) cells and immature SP $CD8^{low}$ cells, found by flow cytometry analysis, recognized to occupy the outer areas of thymic cortex. Furthermore, throughout ontogeny, these thymic areas increase in size, thus reflecting the phenotype of predominant dividing cells and confirming the increased cortex demonstrated morphologically by Herradón et al. (1991) in DCx embryos. Thus, whereas in both 15- and 17-day-old Sham-DCx embryos, most cycling cells are CD8-positive, in DCx-embryos of the same age, they correspond to DN ($CD4^-CD8^-$) cells.

Because the first wave of cell precursors that colonize chicken thymus apparently comes from the intraembryonic mesenchyme of both aortic and paraortic regions described in 4-to-7-day-old chicken embryos (Dieterlen-Lievre, 1992), we analyzed the possible histological changes occuring in these areas of DCx animals. Although a more quantitative analysis is necessary to confirm our merely morphological results, they do not demonstrate notable variations in these cell populations in DCx embryos, suggesting that at these early developmental stages, the lack of major neuroendocrine centers does not influence embryonic lymphohaemopoietic activity. Also, indirectly supporting this view, we did not find significant differences between either the CVI-His-$C_7^+$ or CD28$^+$ cell populations of the 11-day-old thymus from DCx or Sham-DCx embryos.

In summary, DN (CD4$^-$CD8$^-$) cells and immature SP CD8$^{low}$ cells in DCx embryos represent the most abundant thymic T-cell subsets. In addition, they expand but do not differentiate. Accordingly, this stopping of T-cell maturation results in a drastic decline of the following development T-cell stages, mainly DP (CD4$^+$CD8$^+$) cells and TcR αβ-expressing thymocytes.

On the other hand, one of the most remarkable aspects of our results is the lack of important changes in some T-cell subsets, including CD8$^{high}$ cells and TcR γδ-expressing thymocytes of DCx embryos. The lack of variation in CD8$^{high}$ cells, which show only a significant decrease at 15 days of incubation but return to control values on day 17, is not easy to explain because they constitute a heterogeneous cell population. According to our own results about 3–4% of total chicken thymocytes are CD8$^+$ TcR γδ T cells (data not shown). Other intrathymic CD8$^+$ cells presumably correspond to a NK cell population that recirculates in the chicken thymus (Bucy et al., 1989, 1990). The rest of the CD8$^{high}$ cells might be immature cells just reaching the DP (CD4$^+$CD8$^+$) cell compartment. Accordingly, the observed decline in 15-day-old DCx chicks would correspond mainly to this last immature CD8$^{high}$ cell population, and on day 17, the arrival at the thymus of recirculating CD8$^{high}$ NK cells could raise the proportion of total CD8$^{high}$ cells to control values. We cannot discard the possibility, however, that the agents governing T-cell differentiation in DCx-embryos, whatever they are, might affect some cell populations but not others.

This same argument could be applied to the γδ T cells of DCx embryos. However, in this case, it must be borne in mind that γδ T cells differentiate early in the embryonic thymus when the drastic effects of partial decapitation are still not highly evident. Indeed, the chicken γδ cells mature faster than TcR αβ-expressing thymocytes, and on day 15 the first γδ T cells ca be seen in the chicken spleen (Chen et al., 1990; Cooper et al., 1991; our own results).

There were no significant immunohistological variations in the thymic stromal-cell components, suggesting that early partial decapitation affects specifically thymic lymphoid elements. Other authors using the same experimental model reported certain hyperplasia of the thymic epithelial cells (Jankovic et al., 1982; Herradón et al., 1991), but the morphometrical analysis did not demonstrate statistical differences (Herradón et al., 1991). Therefore, we cannot account for the changes of T-cell maturation of DCx embryos by an abnormal condition of the thymic-cell microenvironment. Rather, the quantitative changes observed in the thymic epithelial cells are reflecting the lack of differentiation of T-cell progenitors in DCx embryos. Nevertheless, quantitative and functional studies are necessary to confirm this lack of variation in thymic stromal-cell components of DCx embryos, especially when recent reports in mammals demonstrate conclusively changes in them in the absence of thymocytes (Shores et al., 1991; Surh et al., 1992; Ritter and Boyd, 1993).

We can conclude, therefore, that elimination of major neuroendocrine centres by partial decapitation of early embryonic chicken induces profound and selective changes in the thymic T-cell maturation, including predominantly (1) increase in the number of early T-cell subsets, such as DN (CD4$^-$ CD8$^-$) and immature CD8$^{low}$CD4$^-$ cells; (2) a marked decrease in the number of DP (CD4$^+$CD8$^+$) and TcR αβ + cells; and (3) little variations, by contrast, in the number of mature CD8$^{high}$CD4$^-$ cells and TcR γδ-expressing cells.

Apparently, the maturation of both the first and second wave of cell precursors, which reach the thymic primordium on days 6.5–8 and 12–14 of embryonic life, respectively (Le Douarin and Jotereau, 1975; Coltey et al., 1989), is importantly affected in DCx embryos. As a result, the accumulation of immature T cells causes the increased size of both subcapsulary and outer cortex, whereas the lack of DP (CD4$^+$CD8$^+$) cells and TcR αβ-expressing cells results in the almost total disappearance of the thymic medulla.

The next question to be answered is obviously: What is (are) the factor(s) governing T-cell differentiation in DCx-embryos? Studies in progress in our laboratory (Moreno et al., in preparation) suggest a key role for prolactin in this experimental model, without totally discarding the effects of other hormones, mainly thyroxine, which, as indirectly demonstrated by our current histological study, are lacking in DCx embryos. PRL is one of the first pituitary hormones to be detected in the plasma of chicken embryos (Harvey et al., 1979) and, in mammals, exerts important immunomodulatory influence on the immune system (Gala, 1991; Hooghe et al., 1993).

### MATERIALS AND METHODS

### Embryos and Surgical Procedure

More than 2000 fertile eggs of White Leghorn chickens were purchased from a local supplier and hatched under standard conditions in a forced-draft incubator at 38±1°C and 80% humidity. Embryo age was estimated by the duration of incubation and a minimum of three embryos of studied stage and methodological procedure (light microscopy, immunohistochemistry, RIA, FACS analysis) were used.

At 33–38 hr (stage 10 of Hamburger and Hamilton), chicken embryos were partially decapitated (DCx-embryos) according to Fugo's method (Fugo, 1940). Briefly, a window was opened in the shell and a transversal section was carried out through the midportion of the embryonic prosencephalon. By suctioning, the free anterior portion of the head was then extirpated. The window was closed with adhesive tape and the egg returned to the incubator until sacrifice. The success of the operation was achieved by routine histological sectioning of head. The 33–38-hr embryos, the shells of which were opened and closed, served as control, sham-decapitated (Sham-DCx) animals.

All embryos were inspected daily for viability. Several 4- and 7-day-old DCx and Sham-DCx embryos were fixed *in toto* in Bouin's fixative and embedded in plastic resin for histological analysis of the aortic and paraortic areas. The rest (both DCx and Sham-DCx) were sacrificed on days 9, 11, 13, 15, and 17 of incubation. In each stage studied, chicken embryos were weighed and the largest developed thymic lobes aseptically removed and processed either for immunohistochemical or flow cytometrical analysis (see later). In order to evaluate the endocrinological background for embryos, thyroid, adrenal glands, and gonads were also fixed in Bouin's fixative and embedded in plastic resin for light microscopy examination. Moreover, blood samples were taken from the chorioallantoid membrane of the embryos of the three last developmental stages and the plasma saved for measurement of both PRL and GH levels.

### Immunohistochemistry

Thymic lobes aseptically removed were snap frozen in liquid nitrogen and stored at −80°C until use. Cryosections of 8 μm were fixed in acetone for 10 min and dried overnight. Endogenous peroxidase was blocked with 1% $H_2O_2$ in methanol. After washing in PBS, the sections were incubted 1 hr with the specific mAbs listed in Table 1 and a rabbit anti-mouse Ig conjugated to horseradish peroxidase (Dako, Glostrup, Denmark) diluted 1:40 in PBS, for 1 hr. The peroxidase activity was revealed using 0.05% 3,3′diaminobenzidine (Sigma Co., St. Louis) as chromogen, diluted in PBS plus 0.01% $H_2O_2$. Sections were counterstained with methylene blue and gradually dehydrated with graded alcohol and mounted in DePex. Negative controls were carried out on successive sections that received only the

TABLE 1
Monoclonal antibodies used in this study

| mAb | Dilution | Specificity | Source |
|---|---|---|---|
| CVI-His-$C_7$ | Supern.(FACS) | Pan-leucocytes | S.H.M. Jeurissen, Lelystad, Holland |
| CVI-ChNL-68.1 | Supern. 1:10* | Monocytes/Møs/IDCs | |
| 2–4 | Supern. | CD28 | O. Vainio, University of Turku, Finland |
| 5–5 | Supern. | Immature thymocytes | |
| MUI-83 | Supern. 1:10* | Immature thymocytes | R.L. Boyd, Monash University, Australia |
| MUI-36 | Supern. 1:10* | Møs | |
| MUI-52 | Supern. 1:10* | Cortical epithelial cells | |
| MUI-53 | Supern. 1:10* | Medullary epithelial cells | |
| MUI-55 | Supern. 1:10* | Subtrabecular epithelial cells | |
| CT1 | Supern. | Immature thymocytes | M.D. Cooper, University of Alabama, USA |
| CT3 | Supern. | CD3 | |
| CT4 | Supern. | CD4 | |
| CT8 | Supern. | CD8 | |
| TCR1 | Supern. | TcR αβ | |
| TCR2 | Supern. | TcR γδ | |
| MYC-16 | Supern. 1:2* | MHC/Class II | |

*Dilution used in immunohistochemistry.

second antibody, whereas *in situ* immunostained thymic sections from 2-week-old chickens were used as positive controls. Histological sections were photographed in a Labophot (Nikon) light microscopy provided with an Agfapan APX 100 film (Agfa, Leverkusen, Germany).

### Flow Cytometry

Thymic cells prepared by gently pressing through a steel mesh were suspended in PBS containing 2% FCS and 0.1% $NaN_3$ (pH 7.2). For one-colour analysis, $0.510^6$ cells were incubated with the specific mAbs listed in Table 1 for 30 min, and, after PBS washing, with a FITC-conjugated rabbit anti-mouse Ig (Dako, Glostrup, Denmark) 1:100 diluted in PBS plus 2% FCS. For two-color analysis, one more incubation was achieved with PE-conjugated CT8 mAb.

Cell-cycle analysis was carried out by using 2 x $10^6$ cells fixed for 7 min in 70% alcohol, washed three times in Tris-HCl buffer to pH 6.0, incubated with a RNAse dilution (1 mg/ml Tris-HCl) for 30 min at 37°C, washed in Tris-HCl buffer (pH 6.0), and resuspended in 1 ml of PBS. Finally, 100 µl of a solution of propidium iodide (0.05 mg/ml PBS) was added. In some cases, cells were stained previously either with 2–4 or CT8 mAbs plus FITC-conjugated rabbit and anti-mouse Ig, according to the previously described protocol.

Relative immunofluorescence intensities were measured by flow cytometry with a FACScan (Becton-Dickinson, San Jose, CA). FACScan plus, PC-Lysis, and Cell-Fit softwares were used for analysis of the results.

### Radioimmunoassay

Plasma PRL and GH concentration were measured in 75-µl aliquots by homologous double-antibody RIA with chicken hormones and specific antibodies, kindly supplied by Dr. A. Parlow (Pituitary Hormone and Antisera Center, Harbor UCLA Medical Center, CA). The average plasma PRL and GH values are reported in terms of chicken PRL and GH reference preparations AFP-103228B and AFP-9020C, respectively. Samples were run in a single assay to eliminate interassay variance. The intrassay coefficient of variation was 6%.

### Statistics

In the figures, each datum represents the mean values ± standard errors of percentages of positive cells of, at least, three different experiments. Significant differences were evaluated by Student's $t$ test and differences of $p \leq 0.1$ or $p \leq 0.05$ between control and experimental embryo values are marked as * and **, respectively.

### ACKNOWLEDGMENTS

We thank Drs. Max D. Cooper, C.H. Chen, O. Vainio, R.L. Boyd, and S. Jeurissen for the gift of monoclonal antibodies and Dr. A.F. Parlow for kindly providing RIA reactives. This work was supported in part by CAYCIT grant numbers PM89-0043 and PB91-0374 from the Spanish Ministry of Education and Science.

### REFERENCES

Baroni C. (1967). Thymus, peripheral lymphoid tissues and immunological responsiveness of the pituitary dwarf mouse. Experientia **23**: 182–183.

Baroni C., Fabris N., and Bertol G. (1967). Age dependency of primary immune response in hereditary pituitary dwarf and normal Snell-Bagg mouse. Experientia **23**: 1059–1060.

Besedovsky H., Rey A., and Sorkin E. (1985a). Immunological-neuroendocrine feedback circuits. *In*: Neural modulation of immunity, Guillemin R., Cohn M., and Melnechuk T., Eds. (New York: Raven Press), pp. 163–172.

Besedovsky H., Rey A., and Sorkin E. (1985b). Immune-neuroendocrine interactions. J. Immunol. **135**: 750s–754s.

Betz T.W. (1967). The effects of embyronic pars distalis grafts on the development of hypophysectomized chick embryos. Gen. Comp. Endocrinol. **9**: 172–186.

Bucy R.P., Chen C.H., and Cooper M.D. (1990). Development of cytoplasmic CD3 + /T cell receptor-negative cells in the peripheral lymphoid tissues of chickens. Eur. J. Immunol. **20**: 1345–1350.

Bucy R.P., Coltey M., Chen C.H., Char D., Le Douarin N.M., and Cooper M.D. (1989). Cytoplasmic CD3 + surface CD8 + lymphocytes develop as a thymus-independent lineage in chick-quail chimeras. Eur. J. Immunol. **19**: 1449–1455.

Chen C.H., Bucy R.P., and Cooper M.D. (1990). T cell differentiation in birds. Sem. Immunol. **2**: 79–86.

Chen C.H., Pickel J.M., Lahti, S.M., and Cooper M.D. (1991). Cell surface markers on avian immune cells. *In*: Avian cellular immunology, Sharma J.N. Ed. (Boca Raton, FL: CRC Press), pp. 1–22.

Coltey M., Bucy R.P., Chen C.H., Cihak J., Lösch U., Char D., Le Douarin N.M., and Cooper M.D. (1989). Analysis of the first two waves of thymus homing stem cells and their T cell progeny in chick-quail chimeras. J. Exp. Med. **170**: 543–557.

Cooper M.D., Chen C.H., Bucy R.P., and Thompson C.B. (1991). Avian T cell ontogeny. Adv. Immunol. **50**: 87–117.

Cormier F., and Dieterlen-Lievre F. (1988). The wall of the chick embryos aorta harbours M-CFC, GM-CFC and BFU-E. Development **102**: 279–285.

Cormier F., Paz P., and Dieterlen-Lievre F. (1986). In vitro detection of cells with monocytic potentially in the wall of the chick embryos aota. Dev. Biol. **118**: 167–175.

Covelli V., Jirillo E., and Antonaci S. (1992). Neuroimmune networks and aging of the immune system: Biological and clinical significance. Arch. Gerontol. Geriart. suppl. **3**: 129–144.

Cross R.J., Bryson J.S., and Roszman T.L. (1992). Immunologic disparity in the hypopituitary dwarf mouse. J. Immunol. **148**: 1347–1352.

Dieterlen-Lievre F. (1992). Embryonic chimeras and hemopoietic system development. Bone Mar. Transpl. **9 (Suppl) 1**: 30–35.

Dieterlen-Lievre F., and Martin C. (1981). Diffuse intraembryonic hemopoiesis in normal and chimeric avian development. Dev. Biol. **88**: 180–191.

Duquesnoy R.J. (1972). Immunodeficiency of the thymus-dependent system of the Ames dwarf mouse. J. Immunol. **108**: 1578–1590.

Fugo N.W. (1940). Effects of hypophysectomy in the chick embryo. J. Exp. Zool. **85**: 271–297.

Gala R.R. (1991). Prolactin and growth hormone in the regulation of the immune system. Proc. Soc. Exp. Biol. Med. **198**: 513–527.

Glick B. (1984). Interrelation of the avian immune and neuroendocrine systems. J. Exp. Zool. **232**: 671–682.

Hadden J.W. (1992). Thymic endocrinology. Int. J. Immunopharmac. **14**: 345–352.

Harvey S., Davidson T.F., and Chadwick A. (1979). Ontogeny of growth hormone and prolactin secretion in the domestic fowl (*Gallus domesticus*). Gen. Com. Endocrinol. **39**: 270–273.

Herradón P.G. (1987). Relaciones entre el sistema neuroendocrino y el sistema immune durante la ontogenia. Efecto de la decapitación temprana sobre el desarrollo de los órganos linfoides del embrión de pollo (*Gallus gallus*). Ph.D. dissertation, University of León, Spain.

Herradón P.G., Razquín, B. and Zapata A.G. (1991). Effects of early partial decapitation on the ontogenic development of chicken lymphoid organs. I. Thymus. Amer. J. Anat. **191**: 57–66.

Hooghe R., Delhase M., Vergani P., Malur A., and Hooghe-Peters E.L. (1993). Growth hormone and prolactin are paracrine growth and differentiation factors in the haemopoietic system. Immunol. Today. **14**: 212–214.

Jankovic B.D., Isakovic K., and Knezevic Z. (1978). Ontogeny of the immuno-neuro-endocrine relationship. Changes in lymphoid tissues of chick embryos surgically decapitated at 33–38 hours of incubation. Dev. Comp. Immunol. **2**: 479–492.

Jankovic B.D., Isakovic K., and Micic M. (1982). The thymus-hypophisis interaction in the developing chick embryos: Thymic epithelial cells in hypophisectomized embryos. *In: In vivo* immunology: Histophysiology of the lymphoid system, Nieuwenhuis P., Vander Broek A.A., and Hanna M.G., Eds. (New York: Plenum Press), pp. 343–348.

Jankovic B.D., Isakovic K., Micic M., and Knezevic Z. (1980). Thymus-bursa-hypophysis interaction in the developing chick embryos. *In*: Aspects of developmental and comparative immunology, Solomon J.B., Ed. (New York: Pergamon Press), pp. 529–532.

Jankovic B.D., Isakovic K., Micic M., and Knezevic Z. (1981). The embryonic lympho-neuro-endocrine relationship. Clin. Immunol. Immunopathol. **18**: 108–120.

Johnson B.E., Scanes, C.G., King D.B., and Marsh J.A. (1993). Effect of hypophysectomy and growth hormone on immune development in the domestic fowl. Dev. Comp. Immunol. **17**: 331–339.

Le Douarin N.M., and Jotereau F.V. (1975). Tracing of cells of the avian thymus through embryonic life in interspecific chimeras. J. Exp. Med. **142**: 17–40.

Micic M., Jankovic D.L.J., Isakovic K., and Jankovic B.D. (1983). Forebrain and hypophysis affect development of the bursa of Fabricius in the chick embryo. Period. Biol. **85**: 9s–10s.

Murphy W.J., Durum S.K., Anver M.R., and Longo D.L. (1992a). Immunologic and hematologic effects of neuroendocrine hormones: Studies on DW/J dwarf mice. J. Immunol. **148**: 3799–3805.

Murphy W.J., Durum S.K., and Longo D.L. (1992b). Role of neuroendocrine hormones in murine T cell development: Growth hormone exerts thymopoietic effects in vivo J. Immunol. **149**: 3851–3857.

Ritter M.A. and Boyd R.L. (1993). Development of the thymus: It takes two to tango. Immunol. Today **14**: 462–469.

Shores E.W., Ewijk W.W., and Singer A. (1991). Disorganization and restoration of thymic medullary epithelial cells in T cell receptor-negative scid mice: Evidence that receptor bearing lymphocytes influence maturation of the thymic microenvironment. Eur. J. Immunol. **21**: 1657–1661.

Smith P.E. (1930). Effect of hypophysectomy on the involution of the thymus in the rat. Anat. Rec. **47**: 119–129.

Surh C.D., Ernst B., and Sprent J. (1992). Growth of epithelial cells in the thymic medulla is under the control of mature T cells. J. Exp. Med. **176**: 611–616.

Thommes R.C., Vieth R.L., and Levasseur S. (1977). The effects of hypophysectomy by means of surgical decapitation on thyroid function in the developing chick embryo. Gen. Comp. Endocrinol. **31**: 29–36.

Vainio O., and Lassila O. (1989). Chicken T cells: Differentiation antigens and cell-cell interactions. Crit. Rev. Poultry Biol. **2**: 97–101.

Vogel N.W. (1957). Free tissue cholesterol and growth in chick embryos hypophysectomized by "decapitation". Anat. Rec. **127**: 382.

Weigent D.A., and Blalock J.E. (1987). Interactions between the neuroendocrine and immune systems: Common hormones and receptors. Immunol. Rev. **100**: 77–115.

Woods J.E., Scanes, C.G., Seeley N., Cozzi P., Onyeise F., and Thommes R. C. (1989). Plasma LH and gonadal LH-binding cells in normal and surgically decapitated chick embryos. Gen. Comp. Endocrinol. **74**: 1–13.

